# Promoting Moderate-Vigorous Physical Activity in Overweight Minority Girls

**DOI:** 10.1155/2010/415123

**Published:** 2010-08-01

**Authors:** Norma Olvera, Marilynn Graham, Jessica McLeod, Stephanie F. Kellam, Nancy F. Butte

**Affiliations:** ^1^Department of Health and Human Performance, University of Houston, 3855 Holman Street, Room 104 Garrison Building, Houston, TX 77204-6015, USA; ^2^Children's Nutrition Research Center, Baylor College of Medicine, USDA, 1100 Bates Avenue, Houston, TX 77030, USA

## Abstract

There is limited research on the types of activities that are most effective for promoting MVPA in children. *Purpose*. To assess which types of activities elicit MVPA in overweight minority girls. *Methods*. Sample consisted of 31 overweight (BMI ≥ 85th percentile) Latina and African-American girls (mean age 10.3 ± 1.2 years). Participants wore an Actical accelerometer each day for 8 hours for 15 days to assess engagement in MVPA during their participation in a three-week activity intervention that included traditional fitness, sport skills, games, dancing, and flexibility sessions. *Results*. On average 62% of participants met the MVPA recommended guidelines (60 min/5d/wk) with an average of 68.5 ± 14 minutes of MVPA across the three weeks. Traditional fitness sessions elicited the highest percent of MVPA (mean time spent in MVPA = 32%), followed by dancing and games (mean time spent in MVPA = 21%), sports skills (mean time spent in MVPA = 18%), and flexibility (mean time spent in MVPA = 7%). Step aerobics and rumba fitness elicited the highest proportions of MVPA. *Conclusion*. Traditional fitness activities were identified as the most successful in eliciting MVPA in overweight Latina and African American girls.

## 1. Introduction

Childhood overweight is a major health problem in the United States. The overweight prevalence defined as >85th age- and gender-specific percentile for body mass index (BMI) in children and adolescents aged 2 to 19 years tripled between 1980 and 2002 [1–3]. A more recent estimate from the 2003-2004 National Health and Nutrition Examination Survey suggests that one out of three children in the United States is overweight [4]. The highest rates of overweight prevalence were reported for Mexican American children and African American girls.

Physical inactivity is deemed a major factor contributing to the energy imbalance that leads to excess adiposity [1]. The US Centers for Disease Control and Prevention (CDC) recommend that children and adolescents engage in 60 minutes of daily moderate to vigorous physical activity (MVPA) five out of seven days to achieve health benefits [5]. MVPA levels vary as a function of gender and age. Evidence indicates that only 42% of boys and 11% of girls meet 60 min·d^−1^of MVPA [6]. Similar results have been reported in other studies [7–9]. Pate et al. [10] estimated the percentages of 1379 children and adolescents (aged 7 to 12 years) that met recommended physical activity guidelines using accelerometry. Results of this study revealed that in elementary school aged children exhibited ≥1 hour of physical activity of at least moderate intensity on five or more days of the week. However, just 34% and 25% of adolescent boys and girls, respectively, met this guideline. Troiano et al. [11], using a national sample, reported that 49% of boys and 35% of girls between the ages 6–11 years engaged in the recommended amount of MVPA. However, by adolescence (aged 12–15 years), a significant decline occurred with only 12% of boys and 3.5% of girls achieving this goal. Similarly, data from the National Health and Nutrition Examination Survey 2003-2004 indicated that 52% of boys and 36% of girls between the ages 6–11 years engaged in the recommended amount of MVPA. In contrast, only 15% and 3% of boys and girls, respectively, engaged in MVPA by ages between 12 and 15 years [12]. Overall, these studies indicate that girls are less likely to achieve the MVPA recommendation than boys and with age there is a severe decrease in the percentage of girls achieving the MVPA recommendation.

After controlling for age and gender, overweight children are likely to have lower levels of physical activity than their nonoverweight counterparts [13, 14]. In a study using a national sample, Whitt-Glover et al. [12] found that approximately 60% of normal weight children (aged 6–11 years) achieved MVPA recommendations compared to 31% of overweight children. Similarly, Deforche et al. [15] observed that compared to normal weight, overweight children had lower levels of physical activity. In a study of adolescents, having normal weight-for-age status was significantly associated with higher bouts of moderate to vigorous physical activity [16]. Trost et al. [17] compared physical activity patterns of 133 nonobese and 54 obese sixth-grade children. They observed that over a seven-day period obese children exhibited significantly lower daily accumulations of MVPA relative to their non-obese counterparts. 

Ethnic differences in physical activity levels have also been observed, with Latino and African American children showing lower levels of physical activity than their White counterparts [18–22]. Moreover, among overweight Mexican-American and African-American children only approximately 30% met MVPA guidelines. In a large study of Latino children, Butte et al. [23] observed that overweight adolescent girls had the lowest rates of MVPA. Inactivity in minority children, particularly, among overweight Latina girls, highlights the need to identify which types of physical activities might be effective in promoting the recommended amount of minutes of MVPA among overweight minority girls. Thus, the primary purpose of this exploratory study was to determine which types of physical activity generated greater amounts of MVPA in overweight minority girls. We assessed the contribution of 21 different physical activities (including traditional and nontraditional activities offered in school as well as culturally relevant activities) in eliciting 60 minutes of daily MVPA. These physical activities were grouped into five categories: traditional fitness, dance, games, sports skills, and flexibility. According to the CDC, to achieve MVPA guidelines children and adolescents should engage in aerobic or traditional fitness activities, games, sports skills, and dancing [24]. Based on a compendium of energy expenditure for youth [25], we hypothesized that traditional fitness activities would elicit the highest proportion of minutes of MVPA compared to flexibility sessions, with dance, games, and sport skills falling in the middle. We also tested several types of culturally relevant dances (e.g., rumba fitness, Salsa, and hip hop) since dancing has been identified as effective to increase physical activity in African-American girls [26].

## 2. Methods

### 2.1. Participants

 Thirty-seven girls (27 Latina and 10 African American) participated in this study. They were part of a larger three-week family-based healthy lifestyle summer intervention titled Behavior Opportunities Uniting Nutrition, Counseling, and Exercise (BOUNCE) [27]. Girls' mean age was 10.8 years (SD = 1.2 years, range from 8 to 14 years). Study inclusion criteria included (1) self-identification of Latino or African-American origin from parents and child; (2) age of child between 8 and 14 years; (3) child classified as overweight (body mass index (BMI) ≥85th to 94th percentile for age) or obese (BMI ≥95th percentile for age); (4) a medical examination acknowledging that the child had no physical impediments that could hinder her participation in this study; (5) child's commitment to attend the entire study. Participants were recruited through flyers and referrals by school counselors, nurses, and teachers. Written informed assent and consent from the child and parents were obtained. The University of Houston Committee for the Protection of Human Subjects granted permission for the study to be conducted and approved all research protocols and consent/assent forms.

### 2.2. Intervention

As part of the BOUNCE intervention, girls participated in group sessions of exercise, nutrition education, and behavioral counseling for three weeks, 5 days (Monday–Friday) per week, from 9:00 AM to 5:00 PM each day. A detailed description of the nutrition and behavioral counseling components of the BOUNCE intervention are specified elsewhere [26]. For this section, we will focus on the description of the BOUNCE exercise program. As shown in Table 1, the BOUNCE exercise program was composed of 21 diverse group physical activities of varied target intensities (light 2 METs, moderate 3–5 METs, and vigorous 6 METs and above) according to the Ridley et al. [25] compendium. According to their types, physical activities were grouped into *flexibility, sports skills, games, traditional fitness, and dance* categories. We exposed participants to traditionally (e.g., sport skills) and nontraditionally offered physical activities (e.g., yoga, Pilates, ballet, cheerleading, rumba fitness, Salsa, modern and line dance) in school with the aim to engage participants in diverse, novel, and fun ways to be active.

 The BOUNCE exercise program was standardized with a typical day beginning with a *flexibility* session (30 minutes) followed by a *sports skills* session (60 or 105 minutes) or *games *session (75 minutes). Lunch and a nutrition lesson were then followed by a *traditional fitness *session (60 minutes). Following a counseling session, the day would end with a *dance* session (60 minutes). Thus, participants engaged in four different physical activities daily. Each BOUNCE exercise session included 5-minute warm-up, light to vigorous physical activity, and 5-minute cool-down phases with an emphasis on continuous movement and minimal standing around in an effort to maintain a safe elevated heart rate. For example, participants were encouraged to move or march in place while listening to instructions or waiting their turn. Also, participants were encouraged to engage in the BOUNCE specific physical activities to the best of their abilities. We recognized that despite our best efforts some BOUNCE sessions (e.g., badminton) generated stationary periods among girls.

Instructors certified by the nationally recognized Cooper Institute led exercise sessions at a gymnasium and dance studio located on a university campus. In addition, 3 exercise science undergraduate students assisted instructors and participants during the exercise sessions. For instance, during the exercise session these students mingled with the participants to encourage them with positive praise, to show them how to perform a movement, and to assist them if they were confused or not feeling well. Instructors and exercise assistants participated in four meetings prior to the BOUNCE program to discuss the standardization of the exercise program.

The BOUNCE exercise program was designed to be enjoyable and appealing by allowing participants to use various pieces of exercise equipment (e.g., colorful jump ropes, resistance bands, and hula hoops) and colead some of the exercise sessions, by using several of the participants' favorite music in the exercise sessions, and by partnering participants with others as “buddies.” In addition, we employed other strategies to encourage active participation. First, we asked participants to sign a contract at the beginning of the exercise program by which they agreed to participate in all physical activities. Second, participants received weekly reports of their levels of physical activity achieved in the previous week. Third, prizes were awarded to participants who reached weekly goals. Fourth, participants received handouts on the exercise benefits, components of a healthy lifestyle, and strategies for overcoming barriers to being physically active.

### 2.3. Measures

 Baseline demographic data consisted of questions about age, date and place of birth, and self-described ethnicity. Anthropometric assessments were conducted at baseline and postintervention and included body weight and height measured to the nearest 0.1 kg and 0.1 cm, respectively, using a scale (Tanita TBF 215) and a stadiometer. Height was determined without shoes with the heels of both feet together and the toes pointed slightly outward at approximately a 60-degree angle, arms were at sides, and shoulders were level. Heels, buttocks, and back of the head were touching the vertical backboard and we lowered the headpiece until it firmly touched the crown of the head. BMI was calculated using Quetelet's index (body weight (kilograms)/height^2^ (meters)). BMI values were then used to identify the age- and gender-specific percentile for each child using CDC growth charts [28]. Based on these percentiles, each child was classified as overweight (85th–94th percentile for age and gender), or obese (≥95th percentile for age and gender) [28]. 

Actical accelerometers (Mini Mitter, a Respironics Co., Bend, OR) were used to measure frequency, duration, and intensity of physical activity objectively for 15 days. The Actical is a lightweight accelerometer built from a cantilevered rectangular piezoelectric bimorph plate and seismic mass, which is sensitive to movement in all directions. Actical stores movement information as activity counts. For the proposed study, each participant was shown the placement procedures for the Actical accelerometer at the right hip just above the iliac crest using an elastic strap and plastic buckle to secure the accelerometer around the waist. Participants were instructed to wear the accelerometer daily throughout the BOUNCE intervention (Monday–Friday) from arrival time to the end of the last BOUNCE exercise session. The Actical accelerometer was programmed to collect data from the beginning of the first exercise session at 9:00 AM until the end of the last exercise session at 5:00 PM. The accelerometers were set to record in 60-second epochs. Each day a research assistant logged the start and end times of each physical activity session and recorded the participant's attendance. 

 Upon completion of each five-day intervention week, accelerometer data (activity counts per minute) were downloaded into the Actical program and exported to an Excel spreadsheet for initial analysis. In the initial examination, data completeness was verified against an exercise log and attendance roster. After this initial data screening, activity counts were summed for each day and each activity. Activity counts per minute were partitioned as moderate-vigorous (MVPA: ≥1500 counts·min^−1^), light (LPA: >100–<1500 c·m^−1^), and sedentary activity (SA: ≤100 c·m^−1^) intensities using cutoff points developed by Puyau et al. [29]. The number of daily minutes spent at each intensity level was calculated by averaging the number of minutes spent at each intensity level (e.g., SA, LPA, and MVPA) across all participants for each day. The proportion of total activity time spent in MVPA (number of minutes at or above 1500 counts per minute divided by total time in each activity) was calculated for each of the 21 physical activity sessions. The MVPA achieved during these physical activity sessions was grouped into one of five general activity categories: flexibility, sports skills, games, traditional fitness, and dancing. 

### 2.4. Statistical Analysis

 Descriptive statistics (e.g., means, standard deviations, ranges) were calculated for all variables. The primary study variables included the average number of daily minutes spent in MVPA as well as LPA and SA. Repeated measures ANOVA with repeated contrasts was employed to determine changes in the average daily minutes of MVPA with each successive week of the program (i.e., week 1 to week 2 and week 2 to week 3) and to determine if the percent time spent in MVPA differed significantly among the activity categories. Alpha was set at 0.05. All statistical analyses were conducted using SPSS statistical package, version 15.0.

## 3. Results

### 3.1. Sample Characteristics

 Of the 37 participants, data from six participants were excluded from the analysis because they did not have at least 12 days of accelerometer data reducing the final sample to 31. No significant differences were found between those who completed at least 12 days of accelerometer data and those who did not. Twenty-three percent of the participants were classified as overweight and 77% were classified as obese with a mean (±SD) BMI of 29.2 ± 6.6. The mean number of days that accelerometers were worn by each participant was 13.8 ± 1.1 days. Over half of the girls (59%) came from low-income families as evidenced by receiving federally funded free school meal assistance.

### 3.2. Time (Minutes) Spent in MVPA

 Girls' average time spent in MVPA improved each week (week 1 = 60.47 minutes ± 16.70 minutes, week 2 = 70.32 minutes ± 19.51 minutes, week 3 = 74.70 minutes ± 15.67 minutes). These improvements were statistically significant (*P* < .01) both from week 1 to week 2, and from week 2 to week 3 with an average of 68.5 ± 14 minutes of MVPA across the three weeks of the study. Fifty-six percent of girls met MVPA guidelines during week 1, 66% of girls during week 2 and 63% during week 3 with an average of 62% of the girls meeting MVPA guidelines from 9:00 AM–5:00 PM period when accelerometers were worn.

### 3.3. Proportion of Time Spent in MVPA during Specific Physical Activities

**Figure 1:**
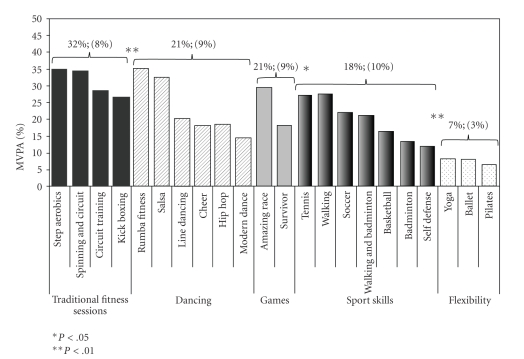
Mean and standard deviations of percentage of time spent in MVPA.

Participants' percentage of time spent in MVPA across each of the different 21 physical activity sessions is presented in Figure 1. The percentage of time spent in MVPA ranged from 6% (Pilates) to 35% (step aerobics and rumba fitness). The average percentage of time spent in MVPA across the five general activity categories (*traditional fitness, dance, sports skills, games, and flexibility*) is also presented. Overall, traditional fitness sessions elicited the highest percent MVPA (mean = 32 ± 8%), followed by dancing and games (mean = 21 ± 9%), sports skills (mean = 18 ± 10%), and flexibility (mean = 7 ± 3%). 

A statistical analysis comparing MVPA across the five activity categories indicated that traditional fitness sessions yielded significantly more MVPA than dancing and games sessions (*P* < .01). The amount of MVPA generated by dancing and games was not significantly different (*P* = .66). However, dancing and games sessions elicited significantly more MVPA than sports skills sessions (*P* = .03), and sports skills sessions elicited significantly more MPVA than flexibility sessions (*P* < .01).

## 4. Discussion

The primary purpose of this exploratory study was to determine which types of physical activities generated the highest proportion of MVPA in overweight minority girls. Findings indicate that traditional fitness activities were the most effective in yielding the highest proportion of MVPA in overweight minority girls while flexibility activities were the least effective with sports skills, dance, and games falling in the middle. An examination of specific physical activities revealed that step aerobics and rumba fitness elicited the highest proportions of MVPA followed by spinning/circuit training and Salsa. In contrast, Pilates sessions elicited the least proportion of MVPA. Thus, these results suggest that it is advisable to offer a variety of traditional fitness activities as well as some culturally appropriate activities such as rumba fitness to elicit MVPA in overweight Latina and African-American girls.

Based on objective measurements of physical activity, it was observed that on average, 62% of participants met MVPA recommended 60 min·d^−1^ guidelines and spent an average of 68.5 ± 14 minutes in MVPA during the 9:00 AM–5:00 PM period when accelerometers were worn. Compared with the previous studies attempting to promote physical activity in minority girls [25, 30–32], our findings are quite noteworthy. In this study, the overweight girls participated in 210–225 minutes of daily physical activity (including approximately 40 minutes of warm-up and cool-down time). Given the fact that not all girls achieved the 60 minutes of MVPA, improvement is needed in motivating overweight girls to increase their PA intensity during the day. This fact not only highlights the need to develop interventions that include effective types of physical activities to achieve MVPA guidelines, but also to train instructors in motivational techniques.

To our knowledge, this is one of the few studies conducted with Latina and African-American girls which assessed MVPA objectively during 15 days of intermittent and sustained physical activity, matched with a valid record of physical activities offered during the intervention. The majority of research to date has focused on total daily minutes of physical activity in a free-living environment and has relied on self-report activity logs [33]. Some limitations of this exploratory study are noted including the small sample size and homogeneity of the sample (overweight and low-income minority girls) which limit the generalizability of the results to the general population of Latina and African-American girls of varied weight and socioeconomic status. Another limitation of this study is the lack of inclusion of psychosocial measures in order to assess participants' enjoyment, motivation, perceived competence, and/or self-efficacy for the activities involved. It would make a contribution to the literature to know if girls engage in more MVPA minutes in activities they claim to enjoy more. For instance, since we did not measure motivation, we could not address whether the highest proportion of MVPA generated by rumba fitness was due to the type of dance movements or due to the greater motivation of the girls or both. Despite the limitations this exploratory study provides valuable information regarding the most effective types of activities in eliciting MVPA in overweight Latina and African-American girls. Such information will guide researchers, physical education teachers, and health educators in designing more effective and culturally appropriate interventions intended to increase the daily amount of MVPA in high risk populations of overweight Latina and African-American girls.

## Figures and Tables

**Table 1:** Weekly offered bounce exercise sessions.

Category	Activity	Target intensity	*Duration (minutes)			
			Week 1	Week 2	Week 3	Total
Flexibility	Yoga/Stretching	Light	30	30	60	120
	Pilates/Stretching		30	30	30	90
	Ballet/Stretching		30	30	30	90

Sports skills	Basketball	Light-moderate	60	0	60	120
	Walking		60	0	0	60
	Soccer		60	60	60	180
	Badminton		0	60	60	120
	Tennis		0	105	0	105
	Self defense		60	60	60	180

Games	Survivor game	Light-moderate	75	0	0	75
	Amazing race		0	75	0	75

Traditional fitness	Step aerobics	Moderate-vigorous	60	60	60	180
	Spinning/circuit		60	60	60	180
	Circuit training		0	60	60	120
	Kickboxing		60	60	0	120

Dancing	Rumba fitness	Light-moderate	60	0	0	60
	Salsa		0	0	60	60
	Hip hop		60	60	60	180
	Cheerleading		60	60	60	180
	Modern dance		0	0	60	60
	Line dancing		0	60	0	60

*Including 5 minutes warm-up and 5 minutes cool-down and instructional times.
